# Dizziness Delaying Discharge: Recognizing Amiodarone-Induced Vestibular Dysfunction as a Reversible Barrier to Inpatient Rehabilitation - A Case Report

**DOI:** 10.51894/001c.165624

**Published:** 2026-07-31

**Authors:** Davids Kupursmits, Harnoor Tokhie

**Affiliations:** 1 University of Michigan Health - Sparrow

## 90

### Background

Amiodarone is a commonly used class III antiarrhythmic agent with an often underrecognized side effect of vestibular toxicity (Sarrazin et al). The impact of amiodarone-induced vestibulopathy on functional recovery in the inpatient rehabilitation facility setting (IRF) has not been previously characterized.

### Case Presentation

We present the case of a 72-year-old male with a medical history of ascending aortic aneurysm status post-surgical repair and atrial fibrillation on amiodarone, who was admitted to IRF for debility secondary to acute appendicitis. During his rehabilitation course, he developed worsening dizziness and balance impairment, limiting participation in therapy and slowing progression toward discharge goals. He felt “swaying or rocking” which worsened with a change in position or ambulation and were different than his previous history of intermittent vertigo. An episode of vertical oscillopsia was also noted. Multiple etiologies were considered and evaluated, including benign paroxysmal positional vertigo (BPPV), stroke, orthostatic hypotension, and persistent postural-perceptual dizziness (PPPD). Neurology and Cardiology were both consulted. MRI Brain revealed no acute stroke. Orthostatic vitals were found to be negative and he also underwent an ILR placement for further arrhythmia monitoring. Further, low vitamin E levels were thought to be contributory and he was started on vitamin supplementation. Ultimately, the patient was diagnosed with PPPD and started on duloxetine with recommendations for outpatient vestibular therapy. However, symptoms persisted and impaired performance in physical therapy and occupational therapy sessions. He was eventually discharged home with ongoing dizziness and required a U-Step walker for ambulation. Following discharge home, amiodarone was discontinued by vascular surgery as an outpatient, with marked improvement in dizziness and gains in ambulation and functional independence within weeks as he began to ambulate without the use of his walker. The resolution of his symptoms and associated improvement of function raise concern for amiodarone-induced vestibulopathy.

### Conclusions

To our knowledge, this is the first documented case of amiodarone - induced vestibular toxicity impairing functional recovery in the IRF setting. This case highlights the importance of systematic medication review in rehabilitation patients with unexplained, persistent dizziness and the need for increased awareness of vestibular toxicity associated with amiodarone use.

